# A Medical College in Asiatic Turkey

**Published:** 1875-03-01

**Authors:** 


### A MEDICAL COLLEGE IN ASIATIC TURKEY.

THE American missionaries in Turkey are wide-awake men, and the widest awake among them is the Rev. Tilman C. Trowbridge, formerly of Michigan.

Mr. Trowbridge is founding a college or university, called the Central Turkish College, in which a prominent feature is to be a Medical Department. This institution is to be located at Aintab, a city some sixty miles north-east of the north-east corner of the Mediterranean Sea. The following extracts from the writings of Mr. Trowbridge, Dr. West, of Sivas, and Dr. Post, Professor of Surgery at Beirut, show the opinions of the men on the ground:

In connection with the Central Turkey College at Aintab, it is proposed to establish a Medical Department for the thorough instruction of young men in all branches of Medical Science. That such a department is greatly needed, and will be eminently useful, there can be little doubt. We invite attention to the following statement, prepared by H. S. West, M.D., a missionary physician residing at Sivas, Asia Minor. Dr. West has lived in Turkey for the past fourteen years; he has had a most extensive practice, and the best possible opportunities to learn the wants of the country in this respect. He is well known in Turkey and in America as an earnest student and a successful practitioner. Since coming to Turkey, he has performed more than a thousand operations for the various diseases of the eye; up to January 1, 1872, he had had ninety-nine cases of *lithotomy*. In other branches of medical practice, he has been equally busy. Probably no man in the Turkish Empire is better qualified than Dr. West to give an opinion in regard to the importance of a Medical School in the interior of the country. He has kindly prepared the following statement of the reasons for the establishment of a Medical School in the interior of Turkey:

I. *Destitution of Educated Physicians in Asia Minor.*—With a population of more than ten millions, the country is almost entirely destitute in this respect. There are a few army physicians stationed at various military posts on account of the soldiers. These men receive an imperfect medical education at Constantinople, where there is a Military Medical School. The other medical practitioners are mostly Armenians, who have never received any professional training, except to be initiated into a routine of practice employed by their ancestors for many generations back, consisting mostly of blood-letting and purging. These men are only found in the principal cities. In the hundreds and thousands of villages there are no medical practitioners whatever. There are no surgical practitioners, except *bone-setters*—ignorant men and women who have learned from their ancestors to apply a bandage, but who have not the least knowledge of anatomy or of any other science. There are also operators for cataract and other diseases of the eye, who travel the country; they perform the old operation of couching. These also, are entirely destitute of education, and know nothing of the eye. The midwives are rude, ignorant women.

II. *Appreciation of Educated Physicians.*—The services of the few European or American physicians, who have been sent out by missionary societies, are eagerly sought, especially in those towns where the people have been accustomed to employ native physicians; and, when the latter have had a very good support, they have shown their preference very manifestly for educated men, and willingness to pay them for their services according to their ability; and whenever medical men have been established, such appreciation has increased from year to year.

III. *Capability of the Natives of Becoming Successful Physicians.*—This has been shown by the fact that some of the leading and most skillful physicians of Constantinople are Armenians, who have been educated in Europe or America; also some American physicians of Asia Minor have trained Armenian students, who have become successful practitioners. The candidates for medical studies would be mostly from among the Armenians. These people, in natural abilities and capabitity of education, are very little, if any, behind those of the civilized countries of the world. There would also be some candidates from among the Greeks, and occasionally from among the Turks.

IV. *Prospect of Applicants to Enter a Medical College.*—This is shown by the fact that three medical missionaries who have received students have had many more applications than they could attend to, and the students have paid well, according to their abilities. All classes have expressed a desire for the opening of such a college in the interior.

V. *The Influence a Medical College would have to Commend the Other Departments of College Education.*—The people see and appreciate at once the practical value of a medical education, and thus learn that of other departments of knowledge. The candidates of the Medical Department would be expected also to be graduates from the Academical, and many would go through the latter for the sake of the former. The connection of the hospital would be of great benefit to the community directly, and as a means of medical education.

VI. *The Aid of the Medical School in Carrying Forward the Missionary Work.*—The advantage medical missionaries possess over clergymen in gaining access to the people is well known. The hold they obtain on the community by their professional services enables them to reach the hearts of many inaccessible to others. Earnest Christians, native physicians, who have been educated by the missionary physicians, have been among the most influential auxiliaries of the missionaries in prosecuting their work.

VII. *The Need of a Medical College for the Turkish-speaking Population of Asia Minor* at the present time is apparent from what has been said. It should be added that there is no prospect of institutions for medical education from governmental or other sources for a long time to come. They should now be opened under Christian auspices. One has already been inaugurated in Beirut, and is doing a good work; but that is for Syria and the Arabic-speaking population. Men are rarely to be found in Asia Minor who can go to Europe or America for medical education. Those who have gone are generally to be found in Constantinople, where there are greater attractions for worldly men. No missionary physicians, with one or two exceptions, are doing anything toward training students, and those who have been trained have been of necessity those who have had but little preliminary education, and will not be able to communicate their knowledge to others. The college will furnish a permanent supply. Taking its candidates from the academical departments, where English as well as other needful studies are taught, it will train men not only to be physicians, but future teachers of medicine, and thus be a permanent institution for good through future generations. At present there are no professional works in the Armenian and Turkish languages. The students of this college will have access to English books and periodicals, and medical literature will be established also in the languages of the land.

We appeal to the members of the medical profession in England and America for aid in organizing this important branch of the college in Central Turkey. The interests of science and civilization will doubtless be greatly promoted by the establishment of this medical school.

